# Clinical and molecular feature-based nomogram model for predicting benefit from bevacizumab combined with first-generation EGFR-tyrosine kinase inhibitor (TKI) in EGFR-mutant advanced NSCLC

**DOI:** 10.1186/s12916-021-02118-x

**Published:** 2021-10-19

**Authors:** Yongchang Zhang, Liang Zeng, Xiangyu Zhang, Yizhi Li, Lingli Liu, Qinqin Xu, Haiyan Yang, Wenjuan Jiang, Analyn Lizaso, Luting Qiu, Ting Hou, Jun Liu, Ling Peng, Nong Yang

**Affiliations:** 1 Department of Medical Oncology, Lung Cancer and Gastrointestinal Unit, Hunan Cancer Hospital, The Affiliated Cancer Hospital of Xiangya School of Medicine, Central South University, Changsha, 410013 China; 2 Graduate Collaborative Training Base of Hunan Cancer Hospital, Hengyang Medical School, University of South China, Hengyang, 421001 Hunan China; 3 Guangdong Lung Cancer Institute, Guangdong Provincial Key Laboratory of Translational Medicine in Lung Cancer, Guangdong Provincial People’s Hospital & Guangdong Academy of Medical Sciences, Guangzhou, China; 4 Department of Medical Oncology, Qinghai Provincial People’s Hospital, Xining, 810000 China; 5 Burning Rock Biotech, Guangzhou, 510300 China; 6 Cancer Center, Union Hospital, Tongji Medical College, Huazhong University of Science and Technology, Wuhan, 430022 China; 7 Department of Pulmonary and Critical Care Medicine, Zhejiang Provincial People’s Hospital, Hangzhou, 310003 Zhejiang China

**Keywords:** Clinical features, Molecular features, Prediction Model, Bevacizumab combined with EGFR-TKI, Advanced NSCLC

## Abstract

**Background:**

The combination of bevacizumab and epidermal growth factor receptor (EGFR) tyrosine kinase inhibitor (TKI) could prolong progression-free survival (PFS) in patients with *EGFR*-mutant advanced non-small-cell lung cancer (NSCLC). Our study investigated the clinical and molecular factors that affect the efficacy of first-generation EGFR-TKI with or without bevacizumab and identify the subset of patients who can benefit from combination therapy.

**Methods:**

Our study included 318 patients with *EGFR*-mutant locally advanced/advanced NSCLC treated with either first-generation EGFR-TKI combined with bevacizumab (A+T; *n* = 159) or EGFR-TKI monotherapy (T; *n* = 159). Two nomogram models to predict PFS and overall survival (OS), respectively, were constructed using two factors that impact EGFR-TKI efficacy: metastatic site and presence of concurrent mutations. The study cohort was stratified into 2 cohorts for training (*n* = 176) and validation (*n* = 142) of the nomogram model. Using the median score from the nomogram, the patients were stratified into two groups to analyze their survival outcome.

**Results:**

The A+T group had significantly longer PFS (14.0 vs. 10.5 months; *p* < 0.001) and OS (37.0 vs. 26.0 months; *p* = 0.042) than the T group. Among the patients with concurrent mutations in tumor suppressor genes, those in the A+T group had significantly longer PFS and OS than the T group (PFS 14.5 vs. 8.0 months, *p* < 0.001; OS 39.0 vs. 20.0 months, *p* = 0.003). The higher scores from the nomograms were associated with the presence of brain/liver/pleural metastasis or concomitant gene mutations, which indicated a higher likelihood of shorter PFS and OS. The validation of the nomogram revealed that patients with lower scores had significantly longer PFS for the T group than those with higher scores (15.0 vs. 9.0 months, *p* = 0.002), but not for the A+T group (15.9 vs. 13.9 months, *p* = 0.256).

**Conclusions:**

Using a nomogram, our study demonstrated that the addition of bevacizumab may enhance the therapeutic effectiveness of EGFR-TKI by overcoming the negative impact of certain clinical and molecular factors on the efficacy of EGFR-TKI.

**Supplementary Information:**

The online version contains supplementary material available at 10.1186/s12916-021-02118-x.

## Background

Lung cancer remains the leading cause of cancer deaths worldwide [1]. Non–small-cell lung cancer (NSCLC) is the most prevalent histological type, accounting for 85% of all lung cancers [2].

Molecular targeted therapies for specific genetic alterations such as epidermal growth factor receptor (EGFR) have significantly improved the progression-free survival (PFS) and overall survival (OS) and profoundly shifted the treatment landscape in recent decades [3–5]. However, most patients who initially respond to EGFR-TKIs will inevitably develop resistance within 1 year [6–8]. To prevent or delay the emergence of acquired resistance to EGFR-TKIs, combination therapy with chemotherapy or antiangiogenic agents plus EGFR-TKIs are an emerging trend and have been evaluated in several clinical trials [9–12]. Bevacizumab is one of the commonly used antiangiogenic monoclonal antibodies that target the vascular endothelial growth factor (VEGF) signaling pathway [13]. Preclinical studies suggested that simultaneous inhibition of the EGFR and VEGF/VEGFR pathways could yield a biologically synergistic effect on antitumor activity and could overcome primary and acquired EGFR-TKI resistance [14, 15]. In the JO25567, NEJ026, and ARTEMIS-CTONG1509 trials, bevacizumab plus erlotinib showed prolonged PFS as compared with erlotinib monotherapy [9–11]. Several other studies also showed that bevacizumab plus EGFR-TKIs significantly prolonged PFS with acceptable toxicity profile than EGFR-TKI monotherapy for patients with *EGFR*-mutant NSCLC [12, 16, 17].

However, data on the effect of concomitant gene mutations at baseline on the clinical efficacy of the EGFR-TKI plus bevacizumab combination therapy are still lacking. Despite treatment with EGFR-TKI, the prognosis remains poor for patients with concomitant gene mutations such as *TP53* [18, 19]. The optimal first-line treatment for NSCLC patients with various concomitant mutations remains unclear. Our previous clinical study revealed that EGFR-TKIs plus bevacizumab was associated with a significantly higher systemic and intracranial objective response rate (ORR) and significantly longer systemic and intracranial PFS [20]. On this foundation, we analyzed the therapeutic effect of the addition of bevacizumab to EGFR-TKI among Chinese patients with *EGFR*-mutant locally advanced/advanced NSCLC. Our main research aim is to evaluate the effect of concurrent gene mutations on patient prognosis. We also constructed two nomogram prediction models for PFS and OS, respectively, based on molecular features such as the presence/absence of concurrent gene mutations, and clinical features such as the specific location of metastasis. We also explored the molecular mechanisms of acquired resistance to the combination therapy.

## Methods

### Patients

All the patients with *EGFR* sensitizing mutation-positive locally advanced/advanced NSCLC treated with either EGFR-TKI monotherapy or EGFR-TKI plus bevacizumab as first-line therapy were retrospectively enrolled in this study for efficacy evaluation and resistance mechanism investigation. The main inclusion criteria included (1) age of 18–75 years, (2) stage IIIB-IV (according to the 8th American Joint Committee on Cancer Staging System), (3) histologically confirmed lung adenocarcinoma/adenosquamous carcinoma with *EGFR* activating mutations (exon 19 deletions or L858R), (4) tumor biopsy sample available at baseline and at progression submitted for next-generation sequencing (NGS) detection for panel of 168 cancer-related genes (Additional file 1: Table S1), (5) received at least two cycles of regimen at standard dosing, and (6) having at least one radiological response evaluation according to Response Evaluation Criteria in Solid Tumors (RECIST) version 1.1. The major exclusion criteria included treatment with other previous systemic therapy. The study protocol was approved by the Hunan Provincial Ethics Committee and written informed consent was obtained from each participant for the use of his or her clinical information for research analysis.

### Study design and treatments

**Fig. 1:**
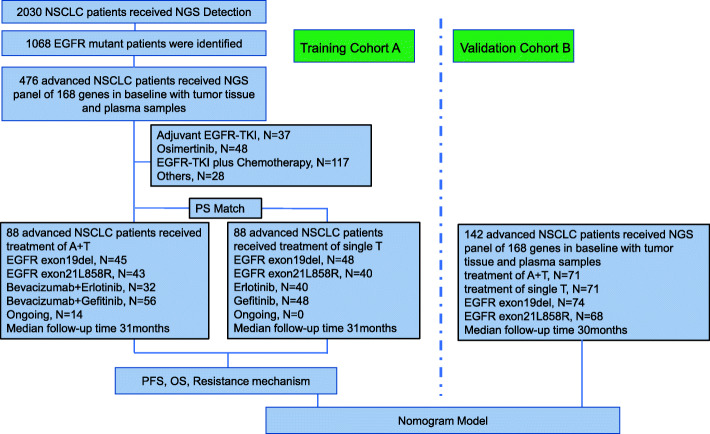
Study flow diagram. EGFR, epidermal growth factor receptor; TKI, tyrosine kinase inhibitor; single T, EGFR-TKI monotherapy; A+T, Avastin (bevacizumab) combined with EGFR-TKI

Our study included a total of 318 patients with *EGFR*-mutant locally advanced/advanced NSCLC who submitted samples for 168-gene panel NGS testing and were treated with either first-generation EGFR-TKI combined with bevacizumab (A+T) or EGFR-TKI monotherapy (single T). For the nomogram model construction, the cohort was further stratified into two groups as training and validation cohorts. The training cohort A was comprised of 176 patients from Hunan Cancer Hospital wherein 88 patients respectively received A+T and T regimens, while the validation cohort B was comprised of 142 patients from Xiangya Hospital wherein 71 patients respectively received A+T and T regimens. To ensure that the two groups had comparable baseline clinical characteristics, the patients included in the T group were selected using propensity score matching with the A+T group using a 1:1 ratio. Retrospective analyses were performed on clinical data, survival outcomes, and mutation profiles at baseline and at progression. Figure 1 illustrates the study design. The data cutoff was August 31, 2020, with median follow-up of 31 months in the training cohort A and 30 months in the validation cohort B. The standard initial dosing in clinical practice is 15 mg/kg, every 21 days for bevacizumab, 150 mg once daily for erlotinib, and 250 mg once daily for gefitinib. Drug dose adjustments were determined by the treating physicians.

### Assessment

Clinical information was collected, including but not limited to demographic characteristics, ORR, PFS, adverse events, and somatic mutation profile. Tumor tissue samples were collected at the initial diagnosis (baseline) and at disease progression by resection or needle biopsy and stored as formalin-fixed paraffin-embedded (FFPE) samples. NGS testing was submitted to Burning Rock Biotech.

### Tissue DNA isolation, capture-based targeted DNA sequencing, and sequence data analysis

Tissue DNA was isolated from FFPE tumor tissue samples using QIAamp DNA FFPE tissue kit (Qiagen, Hilden, Germany). The quality and the size of the fragments were assessed using Qubit 2.0 Fluorimeter with the dsDNA high-sensitivity assay kit (Life Technologies, Carlsbad, CA, USA). NGS library was prepared for each sample. Target capture was performed using a 168 gene panel (Lung Plasma, Burning Rock Biotech, Guangzhou, China), which included 68 lung cancer-related genes and 100 other genes related to cancer development, spanning 0.273 megabases (Mb) of the human genome (Table S1). The indexed samples were sequenced on Nextseq500 (Illumina, Inc., CA, USA) with paired-end reads and average sequencing depth of 1000X. DNA sequence data were analyzed using optimized bioinformatics pipelines based on mapping with the reference human genome (hg19) using Burrows-Wheeler Aligner version 0.7.10, local alignment optimization, duplication marking, and variant calling using Genome Analysis Tool Kit version 3.2 and VarScan version 2.4.3. Single nucleotide variations (SNV) and insertion-deletion variations (INDEL) with population frequency over 0.1% in the ExAC, 1000 Genomes, dbSNP, or ESP6500SI-V2 databases were grouped as single nucleotide polymorphisms (SNPs) and excluded from further analysis. Remaining variants were annotated using ANNOVAR (2016-02-01 release) and SnpEff version 3.6. Analysis of structural variations (SVs) was performed using Factera version 1.4.3. Copy number variations (CNV) were analyzed based on the depth of coverage data of capture intervals. The limit of detection for copy number (CN) gain is CN > 2.25 for hotspot genes (such as *EGFR*, *TP53*, *ERBB2*, and *MET*) and CN > 2.5 for others, while for CN loss is CN < 1.75 for hotspot genes and CN < 1.5 for others.

### Statistical analysis

Median survival was calculated by the Kaplan–Meier method. The Cox proportional hazards model was used for multivariable survival analysis. The variables with a *p* < 0.1 from the univariate analysis were included in the multivariate analysis. Schoenfeld residuals were used to check the proportional hazards assumption. Based on the multivariable analysis, the nomograms were constructed using R version 2.12.1 (The R Foundation for Statistical Computing, Vienna, Austria). The performance of the nomogram was evaluated by calculating the concordance index (C-index). The nomograms were validated using internal and external validation methods. Calibration of the nomogram was performed by comparing the predicted probability and the actual status after bias correction. All *p* values were two-sided, and *p* < 0.05 was considered statistically significant, unless stated otherwise. All procedures were performed using either SPSS software (version 24) or R Studio (version 2.12.1).

## Results

### Patient characteristics

A total of 159 patients, with 88 patients from Hunan Cancer Hospital and 71 patients from Xiangya Hospital with *EGFR*-mutant locally advanced/advanced NSCLC who were treated with A+T were matched to 159 patients who were treated with single-agent T using propensity score matching with 1:1 ratio (Fig. 1). The two groups and cohorts were comparable for baseline clinical characteristics including age, sex, smoking status, Eastern Cooperative Oncology Group Performance Status (ECOG PS) score, tumor node metastasis (TNM) stage, and tumor histology (Table 1). Cohort A also had comparable baseline somatic mutational characteristics (Additional file 2: Figure S1). In cohort A, baseline brain metastasis was detected in 28 (31.4%) patients in the A+T group and 30 (34.1%) patients in the T group (*p* = 0.451). No significant difference was observed for the distribution of other metastatic sites in both cohorts (Table 1).

**Table 1:** Clinical characteristics

	Total	Training cohort				Validation cohort				*P*
		Total	Patients, NO. (%)		*P*	Total	Patients, NO. (%)		*P*	
Characteristic			A+T	T			A+T	T		
NO. of patients	318	176	88	88		142	71	71		
Median age, years (range)	56 (28–83)	56 (28–83)	55.75 (28–74)	57 (31–84)	0.166		56 (26–75)	57 (33–80)	0.412	0.735
Sex										
Male	128 (40.2%)	67 (38.1%)	36 (40.9%)	31 (35.2%)	0.535	61 (43%)	30 (42.3%)	31 (43.7%)	0.768	0.744
Female	190 (59.8%)	109 (61.9%)	52 (59.1%)	57 (64.8%)		81 (57%)	41 (57.7%)	40 (56.3%)		
Smoking history										
Never	232 (73%)	121 (68.8%)	66 (75%)	55 (62.5%)	0.103	111 (78.2%)	56 (78.9%)	55 (75%)	0.925	0.417
Former	86 (27%)	55 (31.2%)	22 (25%)	33 (37.5%)		31 (21.8%)	15 (21.1%)	16 (25%)		
Pathology										
Adenocarcinoma	318 (100)	176 (100%)	88 (100%)	88 (100%)	1	142 (100%)	71 (100%)	71 (100%)	1	1
Squamous carcinoma	0	0 (0%)	0 (0%)	0 (0%)		0 (0%)	0 (0%)	0 (0%)		
ECOG performance status										
0–1	303 (94.3%)	172 (97.7%)	86 (97.7%)	86 (97.7%)	1	131 (92.2%)	65 (91.5%)	66 (92.9%)	0.975	0.896
2	15 (5.7%)	4 (2.3%)	2 (2.3%)	2 (2.3%)		11 (7.3%)	6 (8.5%)	5 (7.1%)		
Brain metastasis										
Yes	102 (32%)	58 (32.9%)	28 (31.8%)	30 (34.1%)	0.862	44 (30.9%)	23 (32.4%)	21 (29.5%)	0.769	0.711
No	216 (68%)	118 (67.1%)	60 (68.2%)	58 (65.9%)		98 (69.1%)	48 (61.6%)	50 (70.5%)		
Bone metastasis										
Yes	160 (50.3%)	88 (50%)	47 (53.4%)	41 (46.6%)	0.451	72 (50.7%)	34 (47.9%)	38 (53.5%)	0.379	0.637
No	158 (49.7%)	88 (50%)	41 (46.6%)	47 (53.4%)		69 (49.3%)	37 (52.1%)	33 (46.5%)		
Liver metastasis										
Yes	40 (12.5%)	21 (11.9%)	12 (13.6%)	9 (10.2%)	0.643	19 (13.4%)	9 (12.6%)	10 (14.1%)	0.563	0.677
No	278 (87.5%)	155 (88.1%)	76 (86.4%)	79 (89.8%)		123 (86.6%)	62 (87.4%)	61 (85.9%)		
Stage										
IIIa/IIIb	9 (2.8%)	5 (2.8%)	1 (1.1%)	4 (4.5%)	0.368	4 (2.8%)	2 (1.1%)	2 (4.5%)	1	0.614
IV	309 (97.2%)	171 (97.2%)	87 (98.9%)	84 (95.5%)		138 (97.2%)	69 (98.9%)	69 (95.5%)		

### Comparison of PFS and OS in A+T vs. T groups

**Fig. 2:**
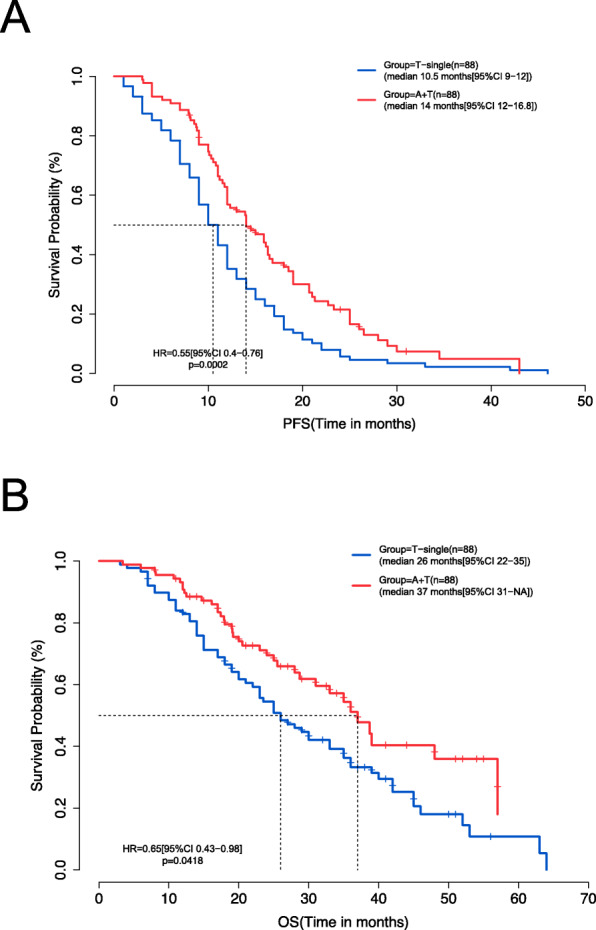
Progression-free survival (PFS) (**A**) and overall survival (OS) (**B**) were significantly longer for patients treated with EGFR-TKI with bevacizumab (A+T) than EGFR-TKI monotherapy (T-single)

The A+T group had significantly longer PFS (median PFS: 14.0 vs. 10.5 months; hazard ratio (HR) = 0.55, 95% confidence intervals (CI) = 0.40–0.76; *p* < 0.001) (Fig. 2A) and improved OS as compared with the T group (median OS 37.0 vs. 26.0 months; HR = 0.65, 95% CI = 0.43–0.98; *p* = 0.042) (Fig. 2B). Mutational status on certain genes, including *TP53* mutation, was associated with significantly different PFS and OS in the T group but not in the A+T group (Additional file 2: Figure S2).

### Impact of metastatic sites on therapeutic efficacy

We evaluated the impact of the brain, liver, or pleural metastasis on the therapeutic efficacy of single T and A+T regimens in the training cohort. In the single T group, the presence of brain, liver, or pleural metastasis was associated with significantly shorter PFS and OS (Additional file 2: Figure S3A). However, in the A+T group, the PFS was comparable for patients with brain, liver, or pleural metastasis, but had poor OS outcome for those with brain/liver metastasis (Additional file 2: Figure S3B).

### Impact of concomitant mutations on therapeutic efficacy

**Fig. 3:**
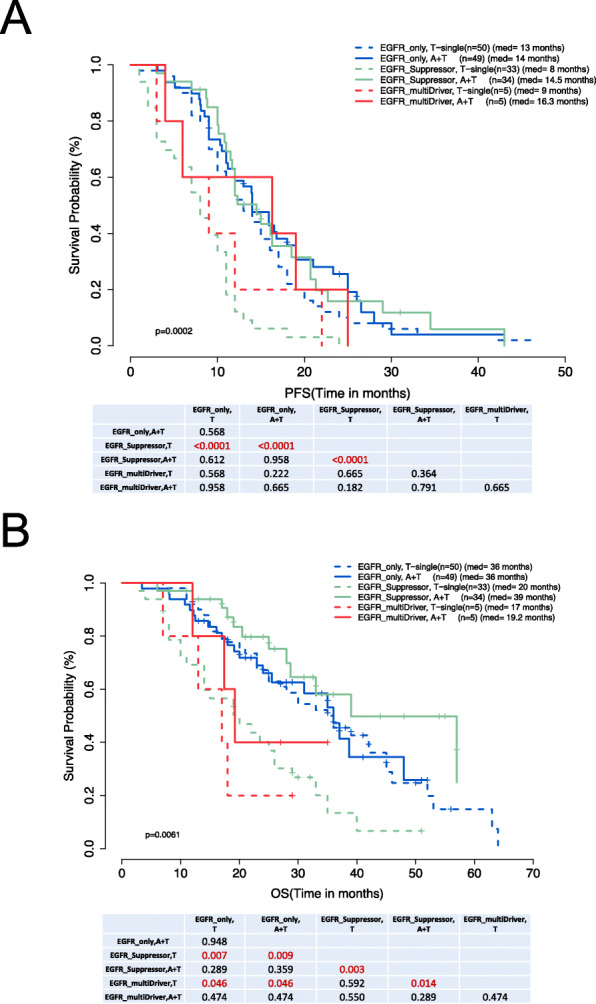
Progression-free survival (PFS) (**A**) and overall survival (OS) (**B**) of patients with *EGFR*-mutant advanced NSCLC without concomitant mutations (EGFR_only) and those with concomitant gene mutations in either tumor suppressor genes (EGFR_Suppressor) or oncogenic driver genes (EGFR_multiDriver) treated with either EGFR-TKI with bevacizumab (A+T) or EGFR-TKI monotherapy (T-single)

We evaluated the impact of the concomitant mutations in tumor suppressor or oncogenic driver genes on the therapeutic efficacy of single T and A+T. Patients with only *EGFR* activating mutations showed comparable PFS with the two treatment regimens (*p* = 0.568) (Fig. 3A). Patients who concurrently harbor mutations in any tumor suppressor gene achieved longer PFS in the A+T group than in the T group (median PFS 14.5 vs. 8.0 months, *p* < 0.001) (Fig. 3A). The patients with concurrent oncogenic driver gene mutations had statistically comparable PFS with A+T and T treatments (16.3 vs. 9.0 months, *p* = 0.665) (Fig. 3A). Similar trends were observed for OS (Fig. 3B).

As the most common genetic alteration observed in our cohort, the presence of *TP53* mutation had a negative impact on the efficacy of single T treatment. Patients who harbored *TP53* mutations had inferior PFS in comparison to those who had wild-type *TP53*; however, it did not impact the efficacy of A+T treatment (Additional file 2: Figure S4A). Expectedly, as compared with single T, A+T significantly prolonged the PFS of *TP53*-mutant patients (median PFS 15.0 vs. 8.0 months, *p* < 0.001), while no difference was observed among *TP53* wild-type patients (Additional file 2: Figure S4A). These observations were also consistent for OS (Figure S4B). As compared to single T, significantly longer PFS and OS were observed with A+T treatment of patients with *TP53* mutations found in exons 5-8 (Additional file 2: Figure S5A-B), but not *TP53* mutations resulting in loss-of-function (Additional file 2: Figure S5C-D) or those with concomitant mutations in *TP53* and *RB1* (Additional file 2: Figure S5E-H).

Furthermore, two patients with stage IV lung adenocarcinoma detected with *EGFR* exon 19 deletion and either concurrent *KRAS* K117N (*n* = 1) or concurrent de novo EGFR T790M (*n* = 1) achieved partial response with A+T regimen, with PFS of > 16.0 months and 10.3 months, respectively. Another patient was detected with concurrent *EGFR* exon 21 L858R concurrent with de novo EGFR T790M and had best response of progression disease and PFS of 1 month with single-agent gefitinib.

### Nomograms

**Fig. 4:**
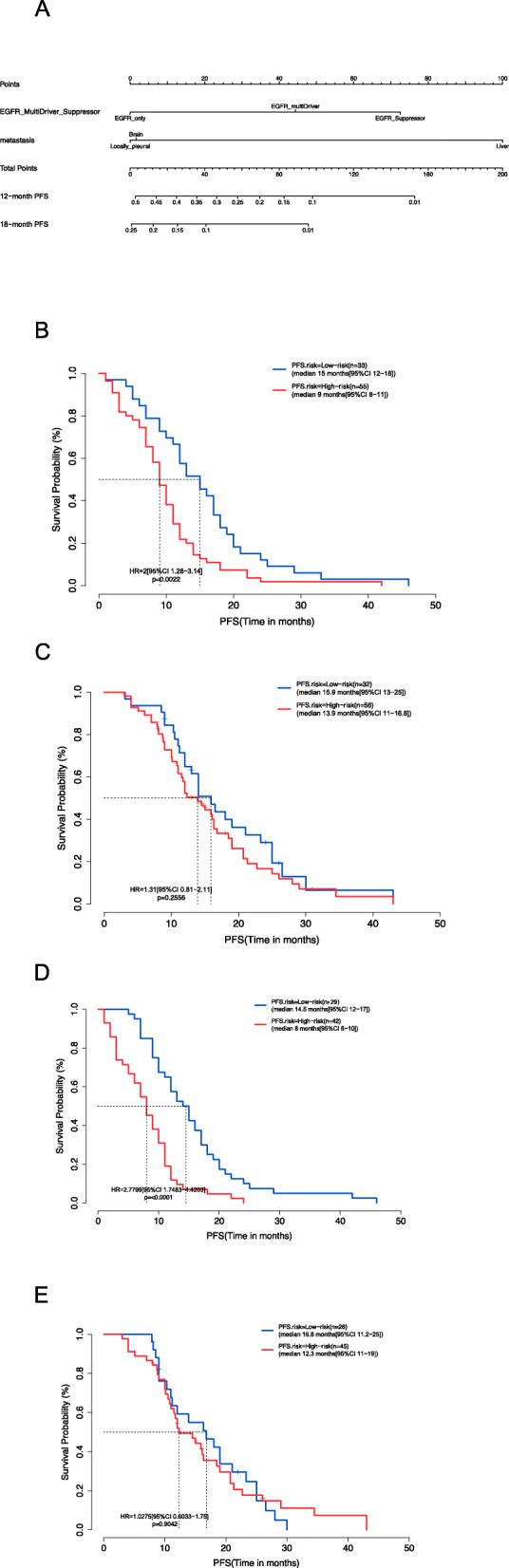
Nomograms were constructed for predicting the risk of 12-month and 18-month progression-free survival (PFS) (**A**). Survival analyses of the training cohort A (**B**, **C**) and validation cohort B (**D**, **E**) were performed using Kaplan-Meier curves to compare the PFS of patients in the T group (**B**, **D**) or A+T (**C**, **E**) group, which were further subgrouped according to the median score into low-risk (< median score) and high-risk (≥ median score)

In training cohort A, through statistical analysis and in consideration of clinical significance, the presence/absence of concurrent genetic mutation (i.e., exclusive *EGFR* activating mutations, concurrent oncogenic driver gene mutation, or concurrent tumor suppressor gene mutation) and the location of metastasis (i.e., locally pleural, brain, or liver metastasis) were selected as the molecular and clinical factors to generate the nomograms for estimating the 12-month and 18-month PFS rates of T treatment (Fig. 4A). As shown in the nomogram, a lower total score/point was associated with having only *EGFR* mutations and/or having only intrathoracic metastasis and indicated a lower risk of disease progression with T treatment, whereas a higher score indicated a higher risk of disease progression due to the presence of concurrent gene mutations and/or presence of brain or liver metastasis. To validate the prognostic utility of the nomogram model, we used the nomogram to score the patients in either the T or the A+T group in the training cohort A or validation cohort B and used the median score as the cutoff to divide the patients as low-risk (< median, low score) and high-risk (≥ median, high score). As expected, patients with lower scores presented with significantly longer PFS than those with higher scores (15.0 vs. 9.0 months, *p* = 0.002) (Fig. 4B). In contrast, the scores from the nomogram were not predictive of PFS with A+T treatment as shown by the comparable PFS between the subgroups (15.9 vs. 13.9 months, *p* = 0.2556), suggesting that the factors included in the nomogram did not impact the efficacy of A+T (Fig. 4C). Consistently, patients with higher scores in the T group of the validation cohort B had significantly shorter PFS than those with lower scores (14.5 vs. 8.0 months, *p* < 0.001) (Fig. 4D), while the patients in the A+T group also had comparable PFS for those with high and low scores (16.8 vs. 12.3 months, *p* = 0.9042), suggesting that the factors included in the nomogram did not impact the efficacy of A+T (Fig. 4E).

Likewise, we constructed a model for estimating the 12-month and 24-month OS of T treatment, using the same molecular and clinical factors included in the nomogram model for PFS (Additional file 2: Figure S6A). Similarly, lower scores predicted significantly prolonged OS in the T group (36.0 vs. 21.0 months, *p* = 0.0051) (Additional file 2: Figure S6B); however, the scores were not predictive of the OS for the patients in the A+T group (38.7 vs. 28.0 months, *p* = 0.1221) (Additional file 2: Figure S6C).

The fact that this model was not applicable for the A+T-treated patients suggested that these molecular and clinical factors negatively impacted the efficacy of T treatment but did not affect A+T treatment. Hence, we further evaluated each variable included in the nomograms on their impact on A+T efficacy using Cox multivariable analysis. We found that none of these factors impacted the PFS for A+T treatment (Additional file 2: Figure S7A), while the presence of brain metastasis (HR = 2.96, 95% CI = 1.51–5.83, *p* = 0.002) and liver metastasis (HR = 2.31, 95% CI = 1.02–5.22, *p* = 0.044) significantly deteriorated the OS of A+T-treated patients (Additional file 2: Figure S7B).

### Resistance mechanism

Resistance mechanisms after disease progression from single T and A+T regimens were only evaluable for 79 and 63 patients included in the training cohort A, respectively.

**Fig. 5:**
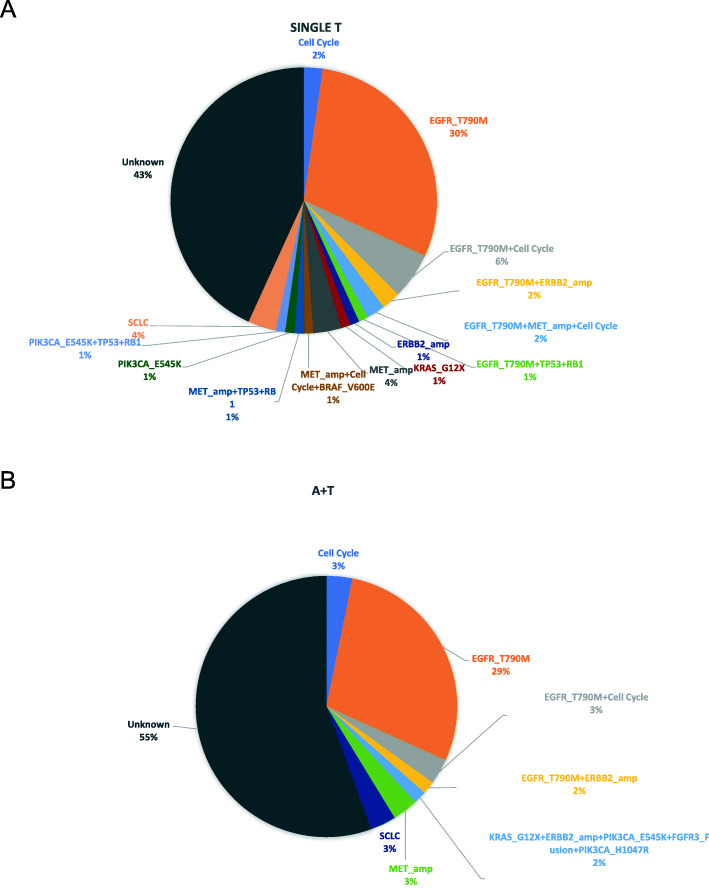
Distribution of acquired resistance mechanism of patients who progressed from **A** EGFR-TKI monotherapy (T) or **B** EGFR-TKI with bevacizumab (A+T)

At progression, *EGFR* T790M was the major resistance mechanism in both groups and was detected from 41% of the T group and 34% of the A+T group. The distribution of acquired mutations in the T group included exclusively *EGFR* T790M (30%), *EGFR* T790M plus cell cycle gene mutations (6%), *EGFR* T790M plus *ERBB2* amplification (2%), *EGFR* T790M plus *MET* amplification plus cell cycle gene mutation (2%), and *EGFR* T790M plus *TP53* mutation plus *RB1* mutation (1%) (Fig. 5A). The distribution of acquired mutations in the A+T group included exclusively *EGFR* T790M (29%), *EGFR* T790M plus cell cycle gene mutation (3%), and *EGFR* T790M plus *ERBB2* amplification (2%) (Fig. 5B). As compared to the A+T group, the T group frequently presented with multiple resistant mutations.

Among the 63 patients who progressed from A+T regimen, three patients were detected with *RB1* mutation wherein two patients (67%) were confirmed with histological transformation to small-cell lung cancer (SCLC). Among the 79 patients who progressed from single T regimen, six patients were detected with *RB1* mutations, of which four patients (67%) were confirmed to have SCLC transformation.

## Discussion

Prior clinical trials have demonstrated the improvement of PFS with the addition of bevacizumab to the EGFR-TKI treatment [9–12, 21]. However, a major challenge remains in the selection of patients who would derive more benefit from the addition of bevacizumab. In addition, the mechanism underlying this improvement is still largely unknown. In this exploratory study, we identified the landscape of the concurrent genomic alterations in *EGFR*-mutant NSCLCs and explored the subset of patients who can benefit from the addition of bevacizumab into first-line EGFR-TKI regimen. Our study demonstrated that the addition of bevacizumab may overcome the clinical and molecular factors that negatively impact EGFR-TKI efficacy and thus enhance its therapeutic effectiveness.

In our study, the median PFS was 14.0 months in the A+T group and 10.5 months in the T group, which were shorter than the reported outcomes of the NEJ026 study (median PFS 16.9 months in erlotinib plus bevacizumab group and 13.3 months in erlotinib alone group) and the ARTEMIS-CTONG1509 (median PFS 17.9 months in erlotinib plus bevacizumab group and 11.2 months in erlotinib alone group [10, 11]. We consider our observation to be acceptable due to the complicated situations in the real-world clinical practice. Interestingly, our study suggested a significantly longer OS in the A+T group than in the T group, which is in contrast to the lack of OS difference observed in the JO25567, NEJ026, and ARTEMIS-CTONG1509 studies [9–11]. We recommend further studies with a larger sample size and longer follow-up to clarify this inconsistency.

The concomitant genetic alterations in either tumor suppressor or oncogenic driver genes, or *EGFR* amplification, were frequently detected in our cohort. Among these genetic alterations, we observed that patients having concomitant mutations in the tumor suppressor genes had longer PFS in the A+T group than in the T group, indicating the favorable role of the combination treatment strategy in patients with concurrent tumor suppressor gene mutations. Previous study proved that harboring concurrent tumor suppressor gene mutation was a negative prognostic factor for EGFR-TKI treatment [22]. Our study further demonstrated that these patients could benefit from the addition of bevacizumab, which to some extent can improve not only the PFS, but also the OS of this subset of patients.

Of the tumor suppressor gene alterations, we observed that *TP53* mutation, which frequently occurs in many human malignancies and is associated with cancer development and progression, was also the most common concurrent genetic alteration in our cohort (41%). This may contribute to the clinical significance of the addition of bevacizumab to EGFR-TKI regimen in our population, particularly among the patients with concomitant mutations in *TP53* or other tumor suppressor genes. Retrospective studies have shown that the presence of mutant *TP53* is associated with poorer PFS with EGFR-TKI therapy [18, 19, 23–25]. Consistent with these studies, our study suggested that concomitant *TP53* mutation negatively impact the PFS for EGFR-TKI monotherapy; however, this was not observed in the A+T group. Besides, compared with EGFR-TKI monotherapy, A+T prolonged the PFS in *TP53*-mutant patients, which was not observed among *TP53* wild-type patients. We speculated that the addition of bevacizumab counteracted the negative effect of *TP53* mutation on EGFR-TKI efficacy and *TP53* mutation may serve as a biomarker for predicting the subset of patients who may benefit from the bevacizumab combined with EGFR-TKI. This conclusion was to some extent supported by some prior studies evaluating the impact of *TP53* mutation on the efficacy of bevacizumab combined with chemotherapy in other solid tumor types including metastatic colorectal cancer and advanced endometrial cancer [26, 27]. Studies have also reported that *TP53* mutation was positively correlated with VEGF-A expression [28].

We constructed nomograms for predicting the risk of progression (PFS) and the risk of death (OS) of the T group based on two significant factors: the presence/absence of concurrent genetic mutations in tumor suppressor genes and oncogenic driver genes and the location of metastasis (locally pleural, brain or liver metastases). Since the models did not impact the PFS or OS for the A+T group, further analyses revealed that the factors included in the nomogram had no effect on PFS of A+T group, while the presence of brain or liver metastasis shortened their OS. This suggested that the factors that negatively impact the efficacy of EGFR-TKI treatment became insignificant when bevacizumab was added to the regimen. Hence, we concluded that the addition of bevacizumab may improve the efficacy of EGFR-TKI through overcoming the negative impact of the clinical and molecular factors which originally affect its efficacy. In the era of precision oncology, other clinical and molecular factors should be taken into account when deciding the treatment strategy. According to the nomogram model, patients with lower scores, or those without concurrent mutation or only have local/pleural metastasis could benefit from single-agent EGFR-TKI regimen. Meanwhile, patients with higher scores, or those with concurrent mutations or have distant metastasis could benefit from A+T combination therapy.

Limitations should be noted in our study. Although the propensity score matching system was used to identify the patients with similar baseline clinical characteristics between the two groups, selection bias was inevitable due to the retrospective nature of our study. In addition, the genomic mutational status was exploratory and had not been verified by another technology. To verify our results, further prospective multi-center studies are recommended.

## Conclusion

In conclusion, we identified that the subset of patients with *EGFR*-mutant advanced NSCLC with concomitant mutations in tumor suppressor genes could derive more survival benefit from the addition of bevacizumab to their EGFR-TKI regimen. Our results also suggest that the addition of bevacizumab may improve the efficacy of EGFR-TKI by overcoming the negative impact of clinical and molecular factors that affect EGFR-TKI efficacy. These observations suggest that the presence of concomitant mutation in tumor suppressor genes could potentially serve as a predictive biomarker for bevacizumab combination therapy. Our study highlighted the importance of interpreting the molecular landscape of the tumor for personalized therapy.

## Supplementary Information

**Additional file 1: Table S1.** The 168 panel genes list.

**Additional file 2: Figure S1.** Baseline mutational profile of Training cohort A. The patients were grouped according to the treatment received; A+T, EGFR-TKI with bevacizumab; T, single-agent EGFR-TKI. **Figure S2.** Heat map illustrating the association between gene mutation or pathway (y-axis) and clinical characteristics, progression-free survival (PFS), and overall survival (OS) (x-axis) for single-agent EGFR-TKI group (A) and EGFR-TKI plus bevacizumab group (B). Blue indicates no statistical difference. The intensity of red color indicates the level of statistical significance with corresponding p-values indicated. **Figure S3**. Patients in cohort A with brain, liver, or pleural metastasis had significantly shorter progression-free survival (PFS) and overall survival (OS) with single-agent EGFR-TKI (A) but not on EGFR-TKI plus bevacizumab combination (B). **Figure S4**. Patients with concomitant *TP53* mutation had significantly longer progression-free survival (PFS) and overall survival (OS) with EGFR-TKI plus bevacizumab combination. Kaplan-Meier curves for PFS (A) and OS (B) of patients with *EGFR*-mutant advanced NSCLC with concurrent *TP53* mutations (TP53+, red color) or wild-type *TP53* (TP53-, blue color) treated with either EGFR-TKI with bevacizumab (A+T, solid lines) or EGFR-TKI monotherapy (T-single, dashed lines). The table below summarizes the p-values. **Figure S5**. Patients with concomitant *TP53* mutations located in exons 5-8 had significantly longer progression-free survival (PFS) and overall survival (OS) with EGFR-TKI plus bevacizumab combination. Kaplan-Meier curves for PFS (A, C) and OS (B, D) of patients with *EGFR*-mutant advanced NSCLC with or without concurrent *TP53* mutations located between exon 5-8 (TP53_hot; A-B) or mutations that result in loss-of-function (TP53_LOF; C-D) and were treated with either EGFR-TKI with bevacizumab (A+T) or EGFR-TKI monotherapy (T-single). Kaplan-Meier curves for PFS (E, G) and OS (F, H) of patients with concomitant *TP53* and *RB1* mutations who were treated with either T-only (E, F) or A+T (G, H). **Figure S6**. Nomograms were constructed for predicting the risk of 12-month and 24-month overall survival (OS) (A). Survival analysis of the training cohort A was performed using Kaplan-Meier curves to compare the OS of patients in the T group (B) or A+T (C) group, which were further subgrouped according to the median score into low-risk (< median score) and high-risk (≥ median score). **Figure S7**. Tabulated summary of the hazard ratios for each molecular and clinical features calculated for the patients treated with EGFR-TKI with bevacizumab (A+T) using Cox multivariable analysis for progression-free survival (A) and overall survival (B).

## Data Availability

The datasets used and/or analyzed during the current study are available from the corresponding author upon reasonable request.

## Abbreviations

A+T: Bevacizumab and EGFR-TKI combination regimen
CI: Confidence intervals
ECOG PS: Eastern Cooperative Oncology Group Performance Status
EGFR: Epidermal growth factor
HR: Hazard ratio
NGS: Next-generation sequencing
NSCLC: Non-small-cell lung cancer
ORR: Objective response rate
OS: Overall survival
PFS: Progression-free survival
T alone: EGFR-TKI monotherapy regimen
TKI: Tyrosine kinase inhibitor
TNM: Tumor node metastasis status
VEGF: Vascular endothelial growth factor
VEGFR: VEGF receptor
