# Supplementary material for: Clinical and molecular feature-based nomogram model for predicting benefit from bevacizumab combined with first-generation EGFR-tyrosine kinase inhibitor (TKI) in EGFR-mutant advanced NSCLC
Source: BMC Med. 2021 Oct 19;19:245. doi: 10.1186/s12916-021-02118-x (PMC8525046; doi:10.1186/s12916-021-02118-x)
Supplement: Supplementary file 1 — Additional file 1: Table S1. The 168 panel genes list. [file 12916_2021_2118_MOESM1_ESM.docx]

**Table S1.** The 168 panel genes list

| **Gene** | **CNV** | **Fusion** | **SNV** | **Gene** | **CNV** | **Fusion** | **SNV** | **Gene** | **CNV** | **Fusion** | **SNV** | **Gene** | **CNV** | **Fusion** | **SNV** | **Gene** | **CNV** | **Fusion** | **SNV** |
| --- | --- | --- | --- | --- | --- | --- | --- | --- | --- | --- | --- | --- | --- | --- | --- | --- | --- | --- | --- |
| *AKT1* | Y | Y | Y | *EGFR* | Y | Y | Y | *JAK1* | Y | Y | Y | *PIK3C3* | N | N | Y | *TP53* | Y | N | Y |
| *ALK* | Y | Y | Y | *EMSY* | N | N | Y | *JAK2* | Y | Y | Y | *PIK3CA* | Y | Y | Y | *TP63* | N | N | Y |
| *APC* | Y | N | Y | *EP300* | N | N | Y | *KDM5A* | N | N | Y | *PIK3CG* | Y | N | Y | *TRIM58* | N | N | Y |
| *AR* | N | N | Y | *EPHA3* | Y | N | Y | *KDM6A* | N | N | Y | *PIK3R1* | Y | N | Y | *TRPC5* | N | N | Y |
| *ARID1A* | Y | N | Y | *EPHA5* | Y | N | Y | *KDR* | N | N | Y | *PMS2* | Y | N | Y | *U2AF1* | N | N | Y |
| *ATM* | N | N | Y | *EPHA7* | N | N | Y | *KEAP1* | Y | N | Y | *POLD1* | N | N | Y | *UGT1A1* | N | N | N |
| *ATR* | N | N | Y | *EPHB1* | N | N | Y | *KIT* | Y | Y | Y | *POLE* | Y | N | Y | *VEGFA* | Y | N | Y |
| *B2M* | N | N | Y | *ERBB2* | Y | Y | Y | *KMT2D* | N | N | Y | *POM121L12* | N | N | Y | *VEGFB* | N | N | Y |
| *BARD1* | N | N | Y | *ERBB3* | N | Y | Y | *KRAS* | Y | N | Y | *PPP2R1A* | N | N | Y | *VEGFC* | N | N | Y |
| *BCL2L11* | N | N | Y | *ERBB4* | N | Y | Y | *LRP1B* | Y | N | Y | *PRKDC* | Y | N | Y | *VHL* | N | N | Y |
| *BCOR* | N | N | Y | *ESR1* | N | Y | Y | *MAP2K1* | N | N | Y | *PTEN* | Y | N | Y | *YES1* | Y | N | Y |
| *BLM* | N | N | Y | *FANCA* | N | N | Y | *MAP3K13* | N | N | Y | *PTPRD* | N | N | Y | *NRG1* | N | Y | Y |
| *BRAF* | Y | Y | Y | *FANCI* | N | N | Y | *MAX* | N | N | Y | *PTPRT* | N | N | Y |  |  |  |  |
| *BRCA1* | Y | N | Y | *FAT3* | N | N | Y | *MCL1* | N | N | Y | *RAD50* | N | N | Y |  |  |  |  |
| *BRCA2* | Y | N | Y | *FBXW7* | Y | N | Y | *MEN1* | N | N | Y | *RAD51B* | N | N | Y |  |  |  |  |
| *BRINP3* | N | N | Y | *FGF19* | Y | N | Y | *MET* | Y | Y | Y | *RAD51C* | N | N | Y |  |  |  |  |
| *BRIP1* | N | N | Y | *FGF3* | Y | N | Y | *MLH1* | Y | N | Y | *RAD51D* | N | N | Y |  |  |  |  |
| *CARD11* | N | N | Y | *FGF4* | Y | N | Y | *MRE11* | N | N | Y | *RAD54L* | N | N | Y |  |  |  |  |
| *CASP8* | N | N | Y | *FGFR1* | Y | Y | Y | *MSH2* | Y | N | Y | *RAF1* | N | Y | Y |  |  |  |  |
| *CBL* | N | N | Y | *FGFR2* | Y | Y | Y | *MSH6* | Y | N | Y | *RARA* | N | Y | Y |  |  |  |  |
| *CCND1* | Y | N | Y | *FGFR3* | Y | Y | Y | *MTOR* | Y | N | Y | *RB1* | Y | N | Y |  |  |  |  |
| *CCNE1* | Y | N | Y | *FLT1* | N | N | Y | *MUTYH* | N | N | Y | *RBM10* | N | N | Y |  |  |  |  |
| *CD274* | Y | Y | Y | *FLT3* | Y | N | Y | *MYC* | Y | Y | Y | *RET* | Y | Y | Y |  |  |  |  |
| *CD74* | N | N | Y | *FLT4* | N | N | Y | *MYCN* | Y | N | Y | *RNF43* | Y | N | Y |  |  |  |  |
| *CDH18* | N | N | Y | *GATA2* | N | N | Y | *NAV3* | N | N | Y | *ROS1* | Y | Y | Y |  |  |  |  |
| *CDK4* | Y | N | Y | *GATA3* | Y | N | Y | *NBN* | N | N | Y | *RUNX1* | N | N | Y |  |  |  |  |
| *CDK6* | Y | N | Y | *GRIN2A* | N | N | Y | *NF1* | Y | N | Y | *SETD2* | N | N | Y |  |  |  |  |
| *CDKN1A* | N | N | Y | *H3F3C* | N | N | Y | *NFE2L2* | Y | N | Y | *SMAD4* | Y | N | Y |  |  |  |  |
| *CDKN1B* | N | N | Y | *HGF* | N | N | Y | *NOTCH1* | N | Y | Y | *SMARCA4* | Y | N | Y |  |  |  |  |
| *CDKN2A* | Y | N | Y | *HIST1H1C* | N | N | Y | *NRAS* | Y | N | Y | *SOX2* | Y | N | Y |  |  |  |  |
| *CHEK1* | N | N | Y | *HIST1H3B* | N | N | Y | *NTRK1* | Y | Y | Y | *SOX9* | N | N | Y |  |  |  |  |
| *CHEK2* | N | N | Y | *HIST1H3G* | N | N | Y | *NTRK2* | Y | Y | Y | *SPOP* | N | N | Y |  |  |  |  |
| *CREBBP* | N | N | Y | *HRAS* | N | N | Y | *NTRK3* | Y | Y | Y | *SPTA1* | N | N | Y |  |  |  |  |
| *CSMD3* | Y | N | Y | *IDH1* | N | N | Y | *PAK5* | N | N | Y | *SRC* | N | N | Y |  |  |  |  |
| *CTNNB1* | Y | N | Y | *IDH2* | N | N | Y | *PALB2* | N | N | Y | *STAG2* | N | N | Y |  |  |  |  |
| *CYP2D6* | N | N | N | *IGF2* | N | N | Y | *PARP1* | N | N | Y | *STK11* | Y | N | Y |  |  |  |  |
| *DIS3* | N | N | Y | *IKZF1* | N | N | Y | *PDGFRA* | Y | Y | Y | *TBX3* | N | N | Y |  |  |  |  |
| *DNMT3A* | N | N | Y | *IL7R* | N | N | Y | *PDGFRB* | N | Y | Y | *TERT* | N | Y | Y |  |  |  |  |
| *DPYD* | N | N | N | *INHBA* | N | N | Y | *PIK3C2G* | N | N | Y | *TGFBR2* | N | N | Y |  |  |  |  |
