# Supplementary material for: Clinical and molecular feature-based nomogram model for predicting benefit from bevacizumab combined with first-generation EGFR-tyrosine kinase inhibitor (TKI) in EGFR-mutant advanced NSCLC
Source: BMC Med. 2021 Oct 19;19:245. doi: 10.1186/s12916-021-02118-x (PMC8525046; doi:10.1186/s12916-021-02118-x)
Supplement: Supplementary file 2 — Additional file 2: Figure S1. Baseline mutational profile of Training cohort A. The patients were grouped according to the treatment received; A+T, EGFR-TKI with bevacizumab; T, single-agent EGFR-TKI. Figure S2. Heat map illustrating the association between gene mutation or pathway (y-axis) and clinical characteristics, progression-free survival (PFS), and overall survival (OS) (x-axis) for single-agent EGFR-TKI group (A) and EGFR-TKI plus bevacizumab group (B). Blue indicates no statistical difference. The intensity of red color indicates the level of statistical significance with corresponding p-values indicated. Figure S3. Patients in cohort A with brain, liver, or pleural metastasis had significantly shorter progression-free survival (PFS) and overall survival (OS) with single-agent EGFR-TKI (A) but not on EGFR-TKI plus bevacizumab combination (B). Figure S4. Patients with concomitant TP53 mutation had significantly longer progression-free survival (PFS) and overall survival (OS) with EGFR-TKI plus bevacizumab combination. Kaplan-Meier curves for PFS (A) and OS (B) of patients with EGFR-mutant advanced NSCLC with concurrent TP53 mutations (TP53+, red color) or wild-type TP53 (TP53-, blue color) treated with either EGFR-TKI with bevacizumab (A+T, solid lines) or EGFR-TKI monotherapy (T-single, dashed lines). The table below summarizes the p-values. Figure S5. Patients with concomitant TP53 mutations located in exons 5-8 had significantly longer progression-free survival (PFS) and overall survival (OS) with EGFR-TKI plus bevacizumab combination. Kaplan-Meier curves for PFS (A, C) and OS (B, D) of patients with EGFR-mutant advanced NSCLC with or without concurrent TP53 mutations located between exon 5-8 (TP53_hot; A-B) or mutations that result in loss-of-function (TP53_LOF; C-D) and were treated with either EGFR-TKI with bevacizumab (A+T) or EGFR-TKI monotherapy (T-single). Kaplan-Meier curves for PFS (E, G) and OS (F, H) of patients with concomitant TP53 and RB1 m [file 12916_2021_2118_MOESM2_ESM.docx]

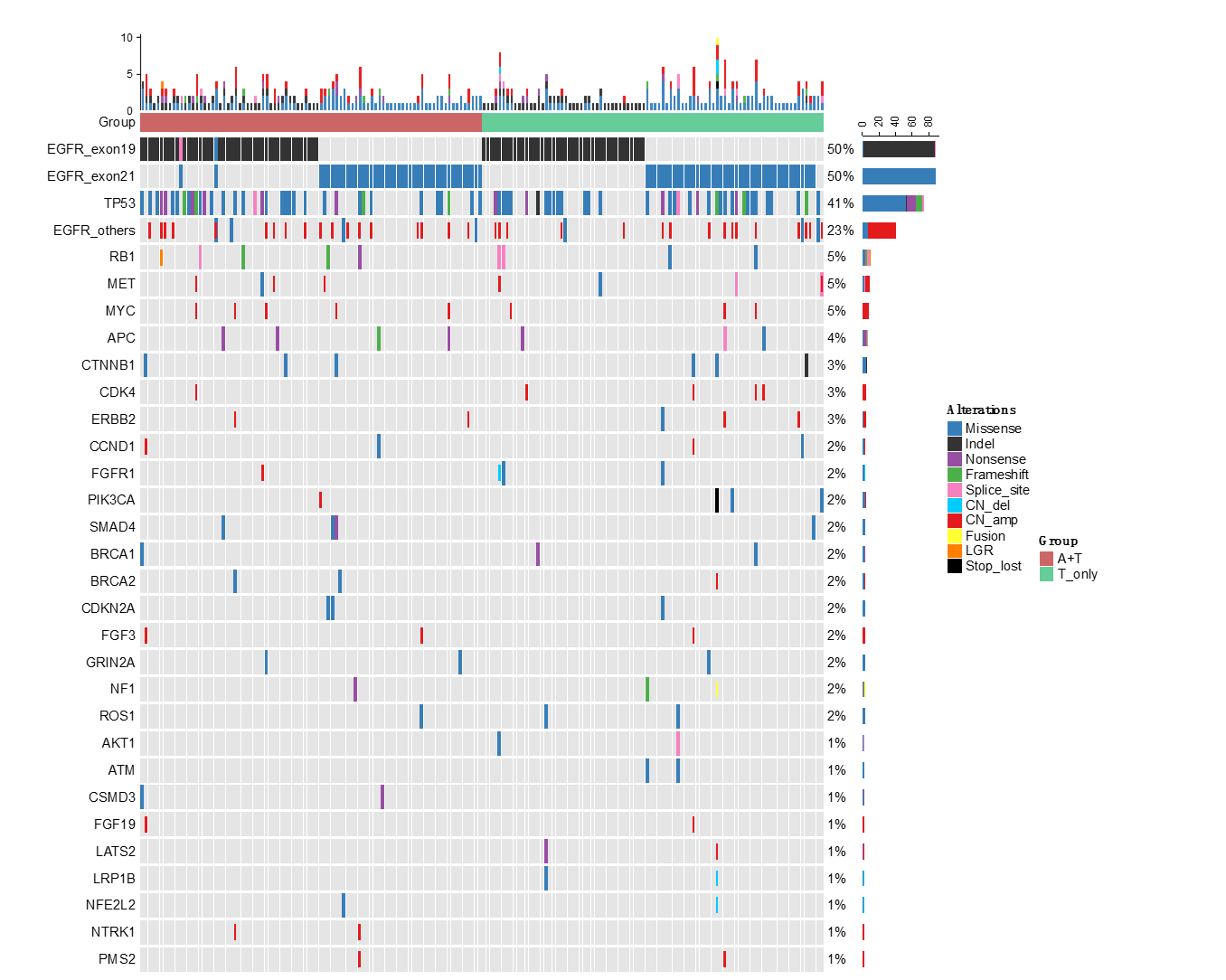


**Figure S1.** Baseline mutational profile of Training cohort A. The patients were grouped according to the treatment received; A+T, EGFR-TKI with bevacizumab; T, single-agent EGFR-TKI.


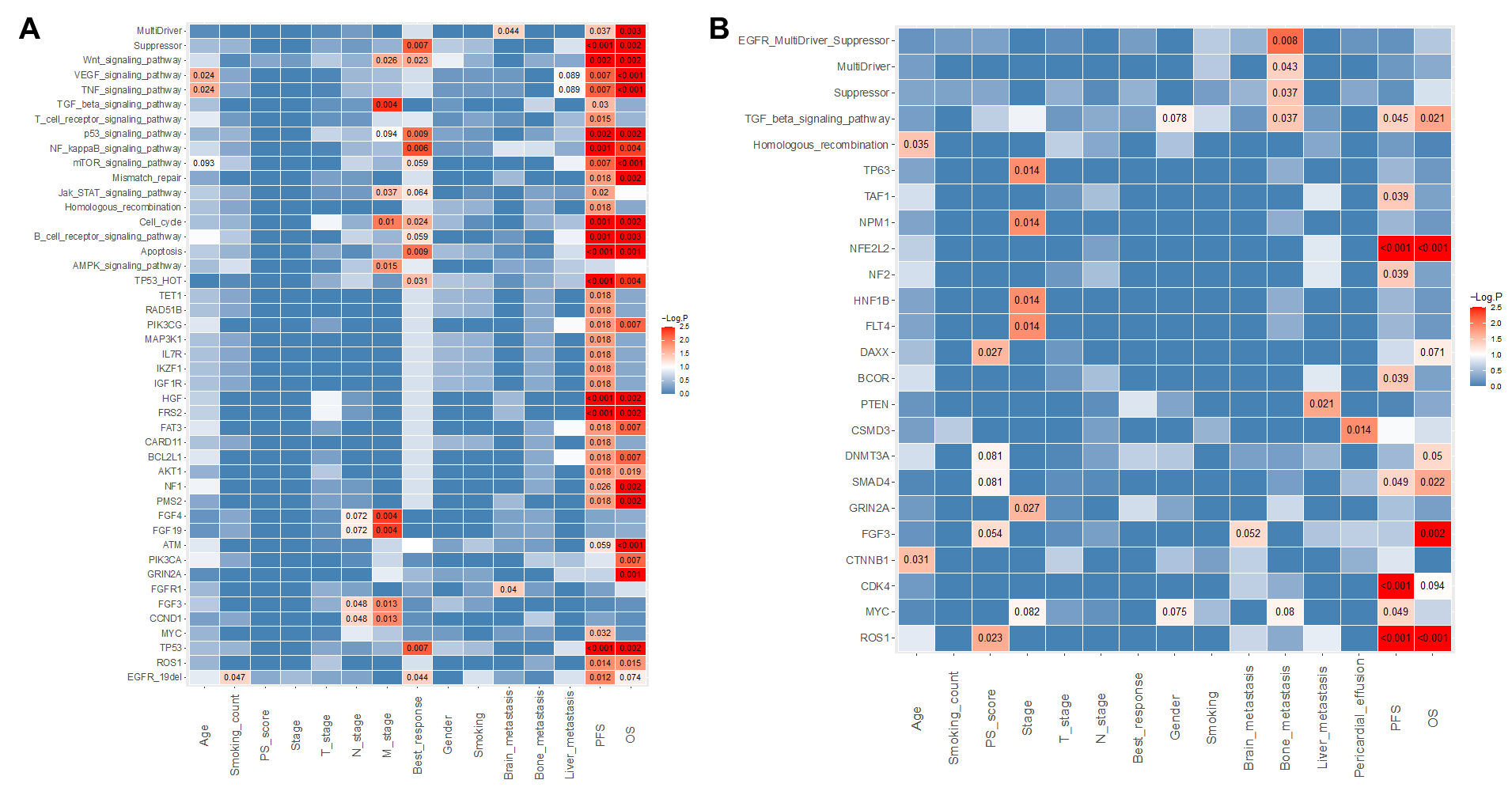


**Figure S2.** Heat map illustrating the association between gene mutation or pathway (y-axis) and clinical characteristics, progression-free survival (PFS), and overall survival (OS) (x-axis) for single-agent EGFR-TKI group (**A**) and EGFR-TKI plus bevacizumab group (**B**). Blue indicates no statistical difference. The intensity of red color indicates the level of statistical significance with corresponding p-values indicated.


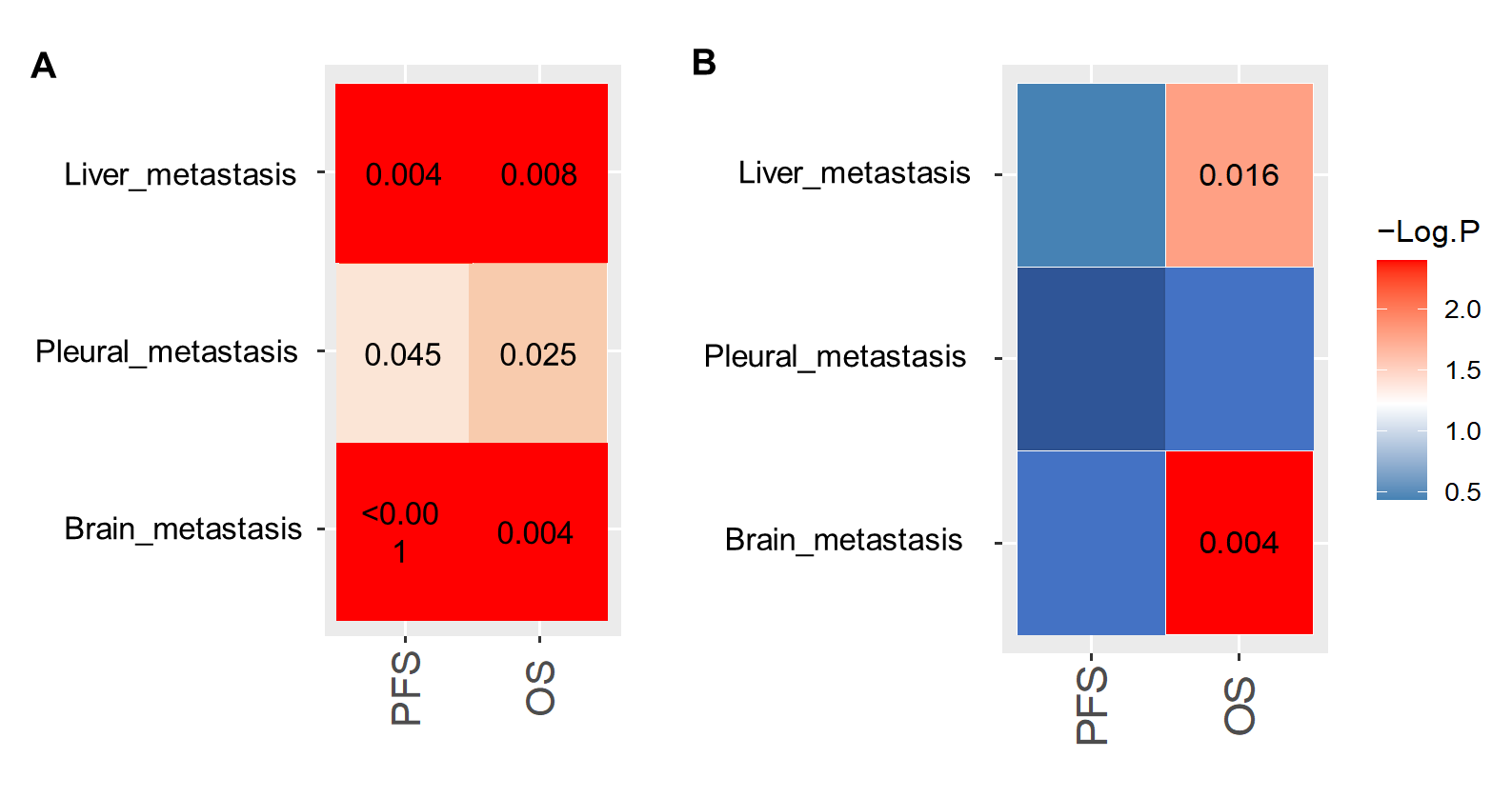


**Figure S3.** Patients in cohort A with brain, liver, or pleural metastasis had significantly shorter progression-free survival (PFS) and overall survival (OS) with single-agent EGFR-TKI (**A**) but not on EGFR-TKI plus bevacizumab combination (**B**).


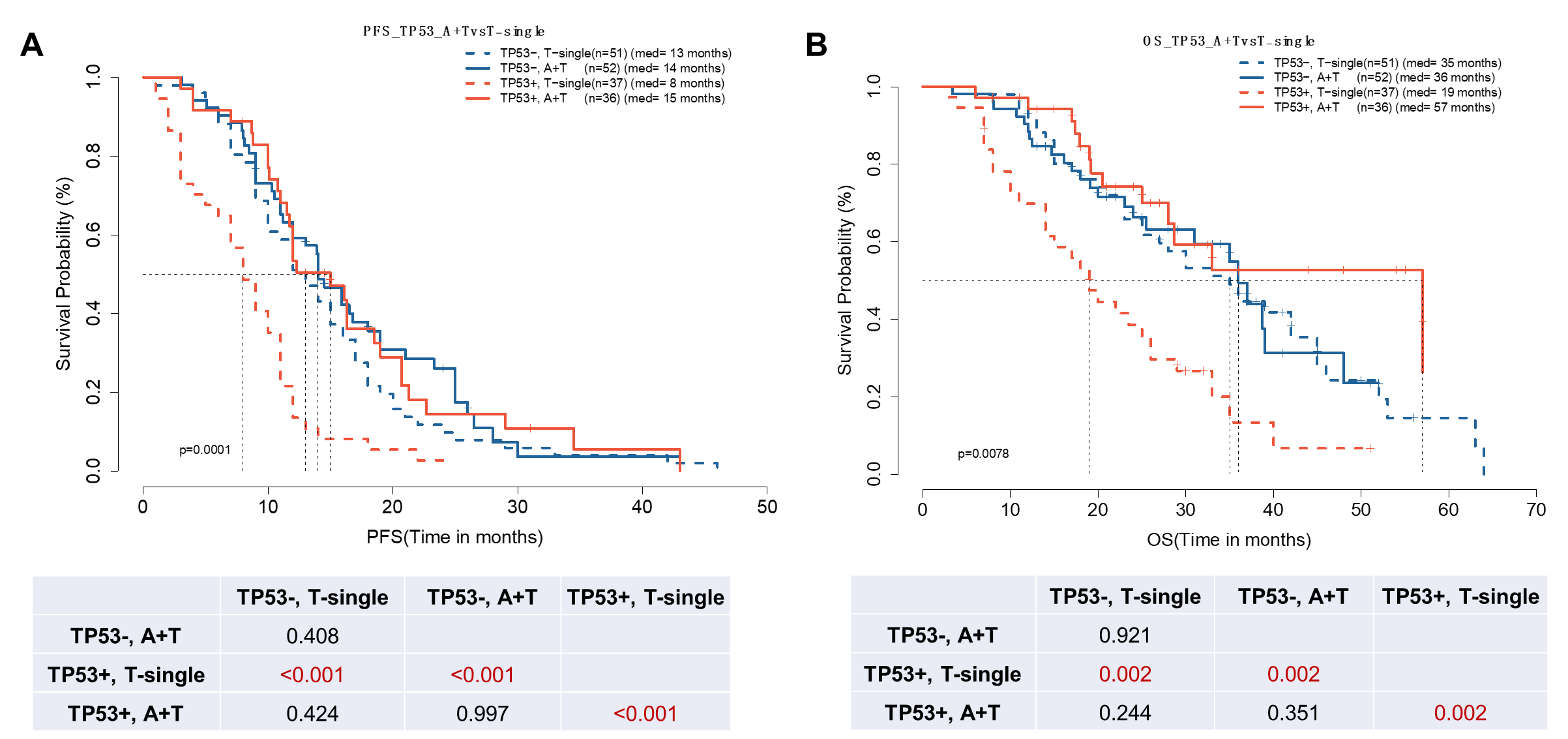
 **Figure S4.** Patients with concomitant *TP53* mutation had significantly longer progression-free survival (PFS) and overall survival (OS) with EGFR-TKI plus bevacizumab combination. Kaplan-Meier curves for PFS (**A**) and OS (**B**) of patients with *EGFR*-mutant advanced NSCLC with concurrent *TP53* mutations (TP53+, red color) or wild-type *TP53* (TP53-, blue color) treated with either EGFR-TKI with bevacizumab (A+T, solid lines) or EGFR-TKI monotherapy (T-single, dashed lines). The table below summarizes the p-values.


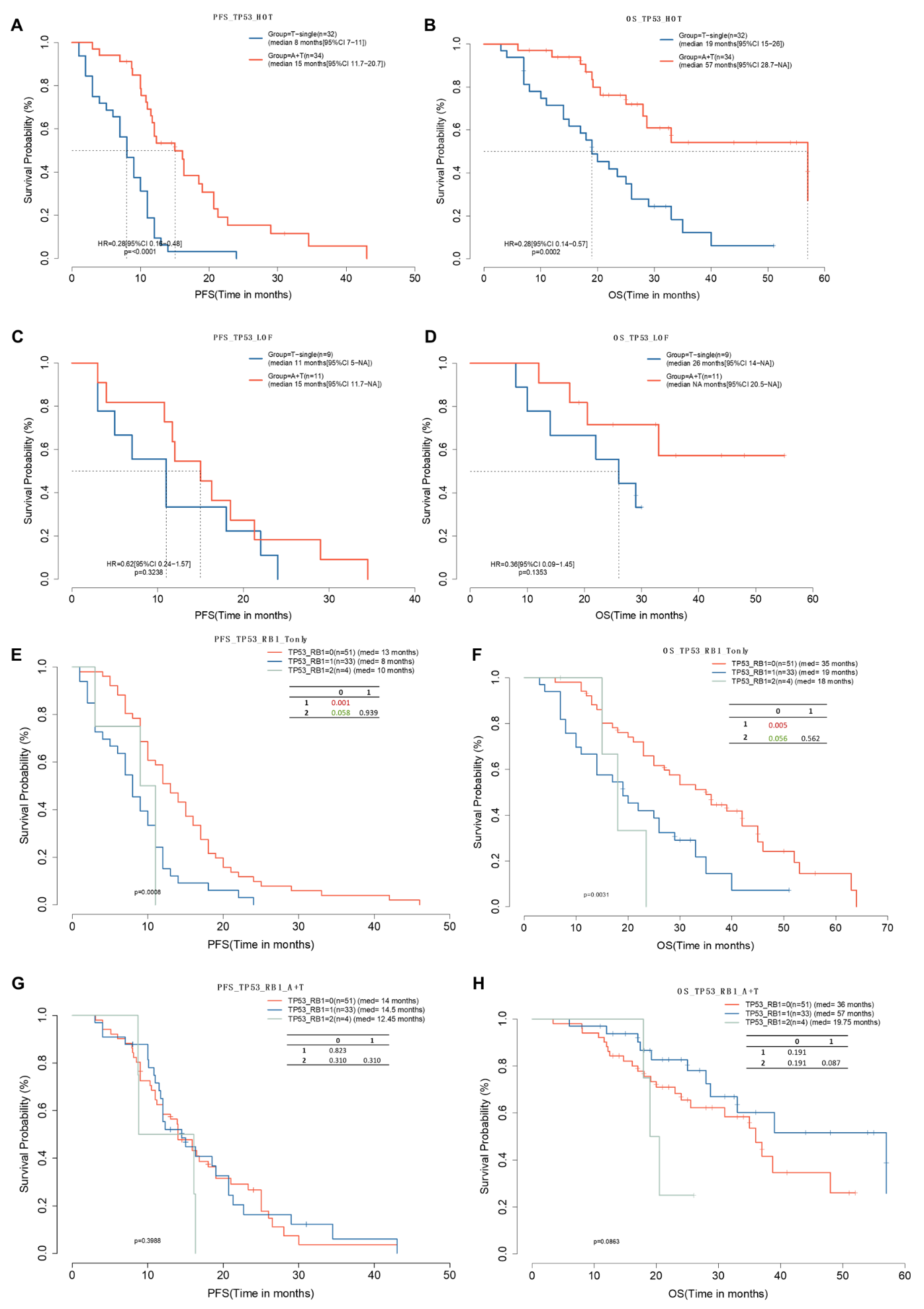


**Figure S5.** Patients with concomitant *TP53* mutations located in exons 5-8 had significantly longer progression-free survival (PFS) and overall survival (OS) with EGFR-TKI plus bevacizumab combination. Kaplan-Meier curves for PFS (**A, C**) and OS (**B, D**) of patients with *EGFR*-mutant advanced NSCLC with or without concurrent *TP53* mutations located between exon 5-8 (TP53_hot; **A-B**) or mutations that result in loss-of-function (TP53_LOF; **C-D**) and were treated with either EGFR-TKI with bevacizumab (A+T) or EGFR-TKI monotherapy (T-single). Kaplan-Meier curves for PFS (**E, G**) and OS (**F, H**) of patients with concomitant *TP53* and *RB1* mutations who were treated with either T-only (**E, F**) or A+T (**G, H**).

**
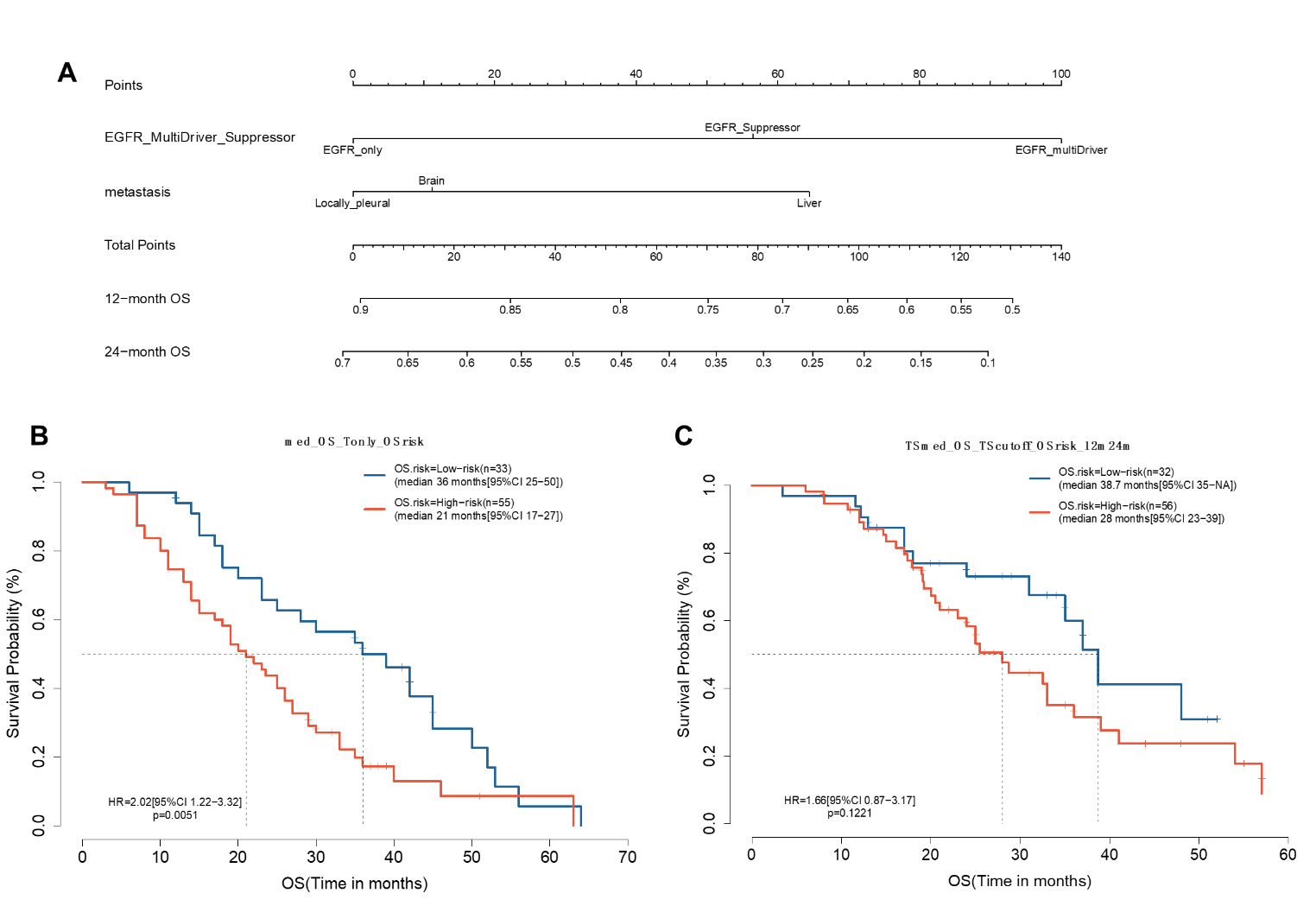
**

**Figure S6.** Nomograms were constructed for predicting the risk of 12-month and 24-month overall survival (OS) (**A**). Survival analysis of the training cohort A was performed using Kaplan-Meier curves to compare the OS of patients in the T group (**B**) or A+T (**C**) group, which were further subgrouped according to the median score into low-risk (< median score) and high-risk (≥ median score).

**
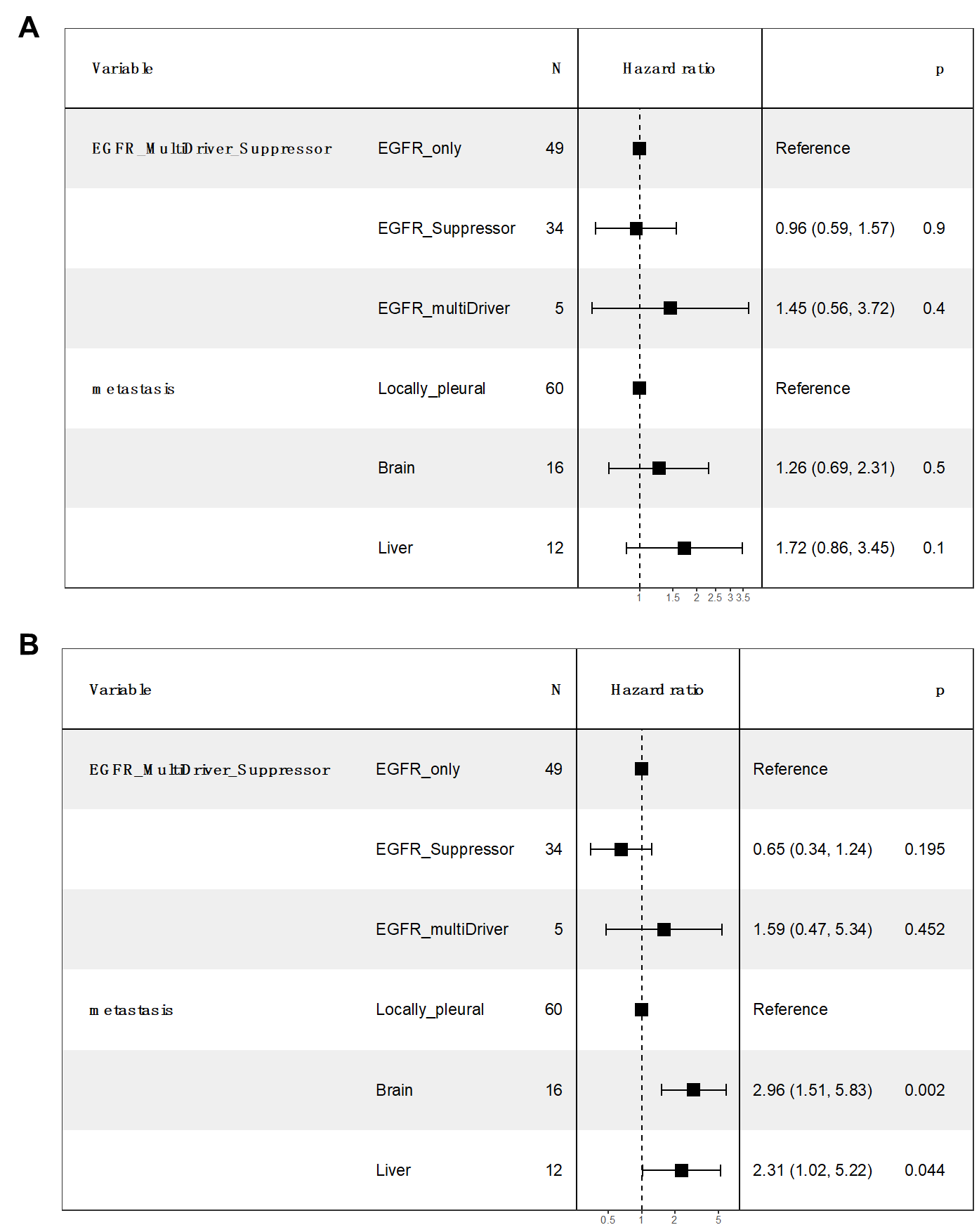
**

**Figure S7.** Tabulated summary of the hazard ratios for each molecular and clinical features calculated for the patients treated with EGFR-TKI with bevacizumab (A+T) using Cox multivariable analysis for progression-free survival (**A**) and overall survival (**B**).
